# Terahertz conductivity mapping of thin films on smart textiles

**DOI:** 10.1038/s41598-024-73113-4

**Published:** 2024-09-25

**Authors:** Alexander Jäckel, Maximilian L. Hupfer, Enrique Castro-Camus, Daniel M. Mittleman, Gabriele Schmidl, Annett Gawlik, Jonathan Plentz, Martin Koch

**Affiliations:** 1 Department of Physics and Material Sciences Center, Philipps-Universität Marburg, Renthof 5, 35032 Marburg, Germany; 2 Department of Functional Interfaces, Leibniz Institute of Photonic Technology (Leibniz IPHT), Albert-Einstein-Str. 9, 07745 Jena, Germany; 3 School of Engineering, Brown University, 184 Hope St., Providence, RI 02912 USA

**Keywords:** Terahertz optics, Electronic devices

## Abstract

Smart textiles that promise to become sensors and actuators for multiple applications are an active area of research. Conductive textiles formed by coating a fabric with a conductive film will play a key role in such applications. Here we present contactless mapping of the terahertz (THz) conductivity of thin conductive films deposited on textiles. These conductivity maps enable non-destructive assessment of the conductivity of such layers and therefore the identification and localization of non-uniformities in local conductivity. The THz measurements are quantitatively consistent with four-point probe measurements of the same areas.

## Introduction

In an increasingly digitalised world, technological advances are constantly changing the way we interact with our physical environment. One of the most exciting areas of development is the combination of electronics and textiles, which has the potential to create a whole new class of wearable technologies^1^. A key aspect of this goal is the development of textile with tailored electrical conductivity, which combine the mechanical properties of conventional textiles with the electrical properties of metals or semiconductor materials^2^. These can be based on conventional textiles made of natural or synthetic materials, which can be functionalised by various coating processes. The textile materials can be coated with metals, conductive oxides, (semi-)conducting polymers or carbon composites^3^. Functional e-textiles with different properties can be created depending on the application, for example as electrodes^4^, thermoelectric actuators^5^, solar cells^6^, electromagnetic shields^7^ or detectors for active laser safety personal protection equipment^8^. Due to the 3D texture and complex surface composition of textile carrier materials, inhomogeneities can occur during functionalisation^9^. In the case of electrodes, these can lead to uneven current distribution or voltage fluctuations, and in the case of electromagnetic shields, to leaky areas. The fragmented nature of the inhomogeneities and the complex structure of the carrier materials make it difficult to characterise these two-dimensional material properties using conventional methods such as four-point probe measurements.

One approach that has been used to map spatially varying conductivity is terahertz time-domain spectroscopy (THz-TDS). This method, which was first demonstrated over 30 years ago, employs short electromagnetic pulses with frequencies in the THz range, propagating in free space, to probe terahertz dielectric properties in a non-contact fashion, via transmission or reflection spectroscopy^10^. From such measurements, it is possible to extract the dielectric properties^11^ or conductivity^12^, even of samples with complicated morphology. By scanning the sample in a raster pattern, two-dimensional conductivity images can be generated^13^, with a spatial resolution determined by the diffraction-limited focal spot size of the focused terahertz beam^14,15^.

In this paper we investigate the homogeneity of the electrical conductivity of textile electrodes. We study textile samples coated with a conductive thin film of two commonly used electrode materials: silver (Ag) and indium-doped tin oxide (ITO). To characterize the homogeneity of conductivity of the samples, we imaged selected areas of both samples using THz-TDS. Based on these THz conductivity images we could localize inhomogeneities in the conductivity of both samples. We found a high correlation between four-point probe measurements of the same areas, suggesting that terahertz imaging is a viable non-contact alternative to the more conventional contact measurement technique.

## Materials and methods

### Samples and sample cleaning

We studied two samples of two different sizes. For this purpose we cut two glass fabrics to act as substrates which were covered with PTFE film. The substrate later coated with ITO had a size of 5 × 5 cm^2^ and the Ag coated substrate had a size of 10 × 10 cm^2^. We chose the thinnest available glass fabrics with a thickness of 70 μm to keep the THz radiation propagation losses caused by the substrate at a minimum. The substrates were soaked in 2 vol% Hellmanex II®(in water), acetone and isopropanol for 15 min each to remove organic residues. To further clean and improve adhesion, the surface of the substrates was treated with oxygen plasma for 15 min.

### Layer deposition

The substrates were coated with 200 nm Ag and 200 nm ITO, respectively. The Ag layers were deposited by electron beam evaporation. The deposition was performed in an oil-free high vacuum system at a pressure of 3.5-${4.0\,\times \,10^{-5}}\,\hbox {mbar}$ with a deposition rate of ${0.8}\,\hbox {nm}\,\hbox {s}^{-1}$ and a heater temperature of ${100}^{\circ }\hbox {C}$. The ITO film with a thickness of ${200}\,\hbox {nm}$ was deposited onto a PTFE foil by DC magnetron sputtering using an ITO 90:10% target, without substrate heating. The argon gas pressure in the deposition chamber was $7\,\times \,10^{-3}\,\hbox {mbar}$. A background pressure of approximately ${1\,\times \,10^{-5}}\hbox {mbar}$ was used. The sputtering power was kept at 70 W resulting in a deposition rate of 0.15 $\hbox {nm}\,\hbox {s}^{-1}$.

### Layer thickness

The thickness of the deposited metals and semiconductors was measured using the Tencor P-7 Stylus Profiler on a nominally identical film deposited on a flat substrate.

### Four-point probe

The sheet resistance was determined using a custom made four-point probe instrument, with gold tips and tip spacing of ${3.5}\,\hbox {mm}$ in a linear configuration. In this way a sheet resistance line profile was determined step by step along the sample, with a minimum resolution determined by this tip spacing. The specific resistance $\rho$ is given by

1 $$\begin{aligned} \rho = d_f G_1 G_2 \frac{U}{I}, \end{aligned}$$

with the voltage *U*, the current *I* and the layer thickness $d_f$. Due to the ratio between sample and measurement geometry, the geometry factors for the sample size $G_1$ = 4.22 and 4.45 were assumed for square samples with a width of 5 and ${10}\,\hbox {cm}$ respectively. For layer thicknesses of 200 $\hbox {nm}$, the geometry factor for the layer thickness size $G_2$ = 1 was used.

### THz-TDS

The THz measurements were performed under nitrogen atmosphere in a transmission setup using a fiber-coupled THz-TDS system. Details about the system can be found in reference^16^ and a general description of THz-TDS can be found in^17^. The sample was mounted on a x-y stage that scanned the sample across the focus of the THz beam pixel by pixel. The scan was performed in ${0.5}\,\hbox {mm}$ steps in both vertical and horizontal directions.

### Data evaluation

To obtain the conductivity $\sigma$ of the film we first evaluated the complex refractive index of the substrate $n_{s}$ by making a transmission image of the bare substrate. Following every fifth pixel of this image we recorded a reference measurement, in order to account for possible slow drifts in the amplitude and temporal position of the generated THz pulse. These reference measurements are performed by moving the sample out of the beam path. We denote the recorded reference signal by $E_{ref}(t)$ and the sample signal by $E_{sam}(t)$. Furthermore, to smooth the signal in the frequency domain we multiplied the signal in the time domain by a Hann window function centered around the main pulse. To then obtain the signals $E_{ref}(\nu )$ and $E_{sam}(\nu )$ in the frequency domain we performed the Fourier transform using the fast Fourier transform algorithm (FFT). The measured transmission coefficient of the substrate $\tilde{t}_s$ is then computed via the ratio $\frac{E_{sam}(\nu )}{E_{ref}(\nu )}$. For each pixel we chose the closest reference measurement with respect to time. The modeled transmission coefficient $t_{s}$ at each frequency $\nu$ is given by

2 $$\begin{aligned} t_{s} = \frac{4n_{s}}{(n_{s} + 1)^2} e^{i \phi _s}, \end{aligned}$$

where $\phi _s = d_{s} n_{s} \omega / c$ is the phase. The variables *i*, *c*, $d_s$ and $\omega =2\pi \nu$ denote the imaginary unit, the speed of light in vacuum, the substrate thickness and the angular frequency of the THz radiation, respectively. We utilize this relatively simple model which only describes a single pass of the pulse through the substrate. It therefore does not account for potential Fabry-Pérot reflections in the signal since we do not expect them to be temporally resolvable due to the low substrate thickness of 70 ${\upmu \hbox {m}}$. Finally, the complex refractive index for each pixel of the substrate image is obtained by varying the value of $n_{s}$ at each frequency in order to minimize the sum of the squared residuals^18^, given by

3 $$\begin{aligned} S_s = (|t_{s}|-|\tilde{t}_{s}|)^2 + (\arg (t_{s})-\arg (\tilde{t}_{s}))^2, \end{aligned}$$

where $\arg (z)$ denotes the phase of the complex number *z* and |*z*| its magnitude. The magnitude of the transmission coefficient is later referred to as the amplitude transmission.

Analogously to the procedure for the bare substrate, we obtained the measured transmission coefficient $\tilde{t}_f$ of the film-substrate sample by making a transmission image of the substrate after it had been coated with the thin film. To then obtain the conductivity of the film, we modeled the transmission coefficient $t_f$ using a transfer matrix description of the complete two-layer sample. Further details about the transfer matrix method (TMM) can be found in reference^19^, which is equivalent to other standard methods for the extraction of the multi-layer complex dielectric parameters from THz-TDS data^20^, with the advantage that it is easier to implement computationally. Based on this model $t_f$ can explicitly be expressed as

4 $$\begin{aligned} t_f = \frac{t_{12}t_{23}t_{31} e^{i (\phi _f + \phi _s)}}{1 + r_{12}r_{23} e^{i 2\phi _f} + r_{31}r_{23} e^{i 2\phi _s} + r_{12}r_{31} e^{i 2(\phi _f+\phi _s)}}, \end{aligned}$$

where $t_{ij}$ and $r_{ij}$ denote the complex Fresnel transmission and reflection coefficients at normal incidence, respectively. Here, the subscripts indicate medium 1 (air), medium 2 (the substrate textile) and medium 3 (the thin film), which was fixed to have a thickness of $d_f={200}\,\hbox {nm}$, and this is the nominal deposited value for both samples.

**Figure 1:**
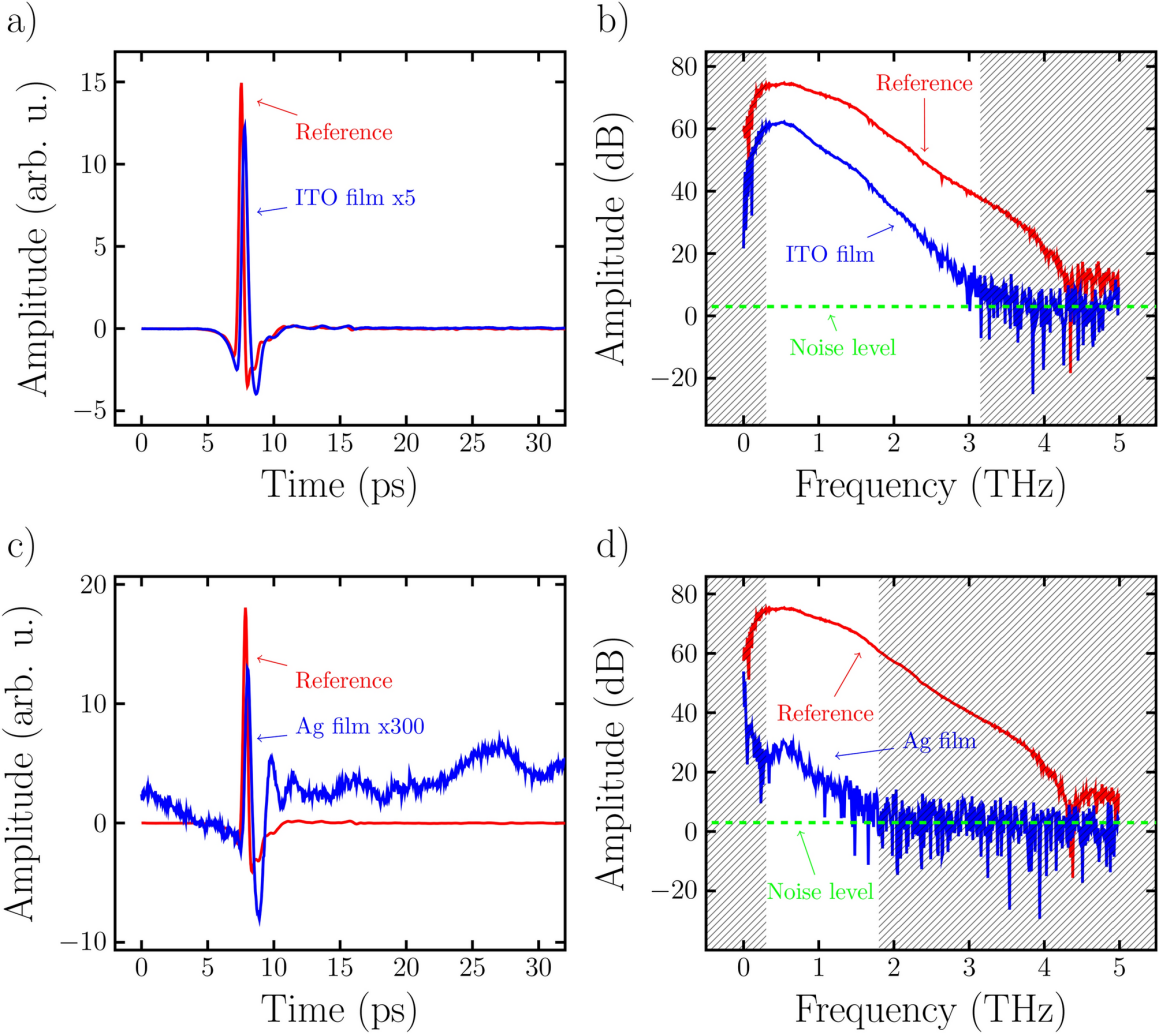
Exemplary waveforms representing a single pixel of the Ag and ITO images compared to two individual reference measurements. (**a**,**c**) Shows the signals in the time domain while (**b**,**d**) show the frequency domain representation of the signals.

As an initial approximation we assumed a Drude conductivity in the investigated frequency range of the thin Ag and ITO films since this has been demonstrated to be a reasonable description of the two materials in previous studies^10,21–23^. The complex refractive index of the thin film $n_f$ can then be related to the conductivity using

5 $$\begin{aligned} n_f = (1+i)\sqrt{\sigma _{DC} / (2\omega \epsilon _0)}, \end{aligned}$$

where $\epsilon _0$ denotes the vacuum permittivity. The model therefore only contains the conductivity as a free parameter since at normal incidence the Fresnel coefficients only depend on the refractive indices of the two layers $n_f$ and $n_s$ where $n_s$ is known from the substrate image evaluation and $n_f$ is given by the conductivity $\sigma _{DC}$ through Eq. (5). Finally we obtained the conductivity by minimizing the summed squared residuals $S_f$ for $\sigma$ given by the expression

6 $$\begin{aligned} S_f = \sum _i (|t_f(\sigma _{DC}, \nu _i)| - |\tilde{t}_f(\nu _i)|)^2, \end{aligned}$$

where $\tilde{t}_f$ is the measured transmission coefficient which is calculated analogously to $\tilde{t}_s$. Figure 1 shows two reference measurements as well as two sample measurements of the ITO and Ag samples in the time (a), (c) and frequency (b), (d) domain, respectively. We excluded the ranges of the spectrum where the amplitude fell below 3 dB corresponding to a signal-to-noise ratio of approximately 1.4. This resulted in a cutoff frequency for the ITO and Ag measurements of approximately ${3.0}\,\hbox {THz}$ and ${1.8}\,\hbox {THz}$, respectively. At the lower end of the spectrum we set the cutoff frequency to ${250}\,\hbox {GHz}$ due to the higher noise level in that region. These ranges are indicated by gray shaded areas in the frequency domain plots. We therefore performed the summation given in equation 6 over the frequencies between the gray shaded areas for the two samples individually.

## Results and discussion

**Figure 2:**
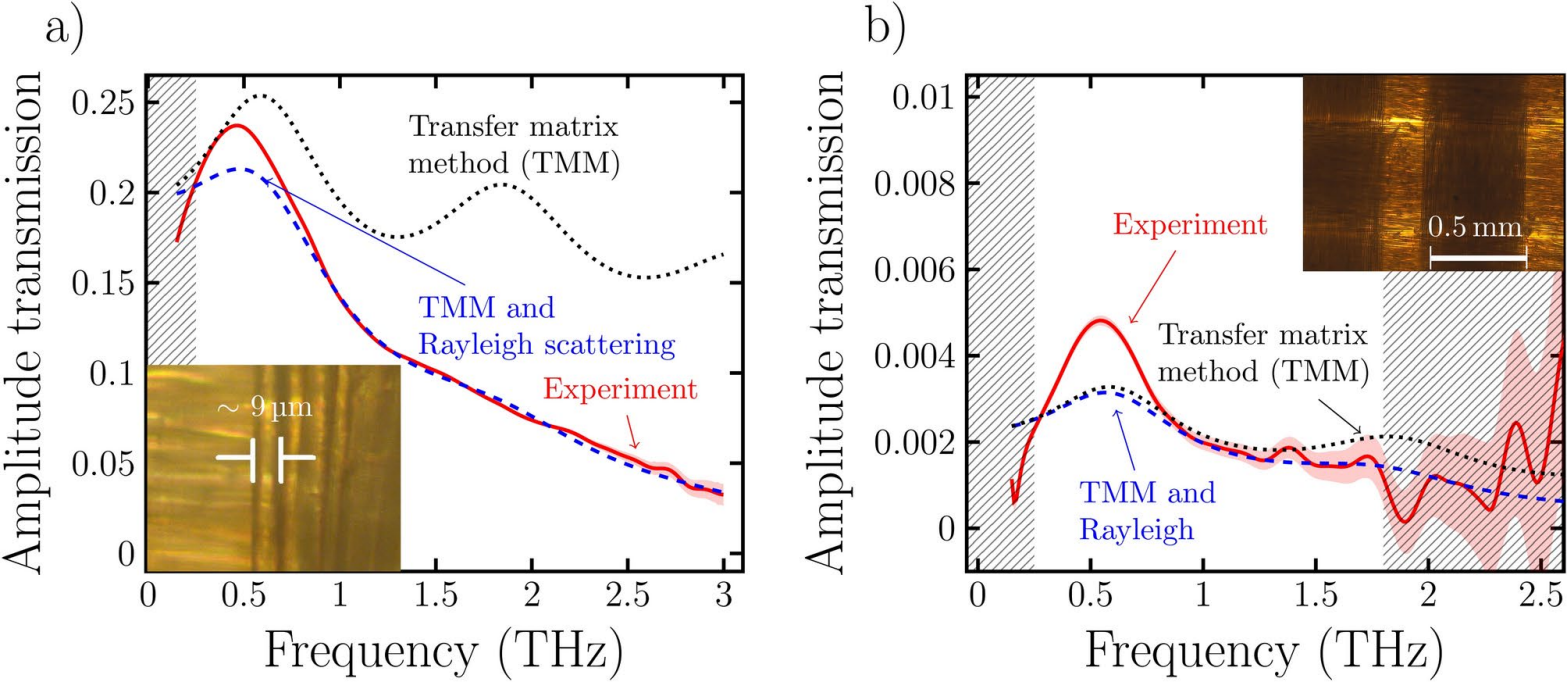
Measured and modeled amplitude transmission shown for ITO and Ag in (**a**,**b**), respectively. The gray shaded regions indicate the frequency regions with a low signal to noise ratio, which were therefore excluded in the fitting procedure. Scaling the Fresnel coefficients of the model by a Rayleigh scattering factor results in a good fit of the data in the region where the data can be trusted. The curves denoted ’TMM’ and ’TMM and Rayleigh’ represent equation (4) with and without the scattering factor, respectively. Insets (**a**,**b**) show a 50x and a 10x magnified microscopic image of the substrate, respectively.

We first imaged the bare substrate before the conductive layer had been applied to the surface. Evaluation of the substrate image resulted in an average refractive index of $1.72\pm 0.03$ and an extinction coefficient of $0.06\pm 0.01$ at ${1}\,\hbox {THz}$ with a low dispersion between ${0.5}\,\hbox {THz}$ and ${3.0}\,\hbox {THz}$ (not shown). Here the error intervals represent the standard deviation from averaging across all pixels of the image. Subsequently, we imaged the same areas of the substrates after the conductive layer deposition. Furthermore, the measured amplitude transmission for a selected point on both samples is shown in Fig. 2. We found that especially in the case of the ITO coated sample the amplitude transmission showed a strong frequency dependence while it was less pronounced for the Ag sample. However, based on measurements and the Drude model it has been shown that for both materials in the thin film geometry a constant conductivity with respect to frequencies below approximately ${3.5}\,\hbox {THz}$ can be expected^21–23^. This in turn means that we would expect the amplitude transmission to be only slightly frequency dependent in contrast to the experimental observation.

In the figure the curve denoted ’transfer matrix method’ represents the calculated amplitude transmission given by Eq. (4). A comparison of the mathematical model to the measured amplitude transmission shows a significant difference which increases with frequency. This deviation could be explained by a frequency dependent Rayleigh scattering factor caused by the fabric structure which would imply a stronger attenuation for higher frequencies. Significant scattering is also to be expected due to the microscopic periodic structure of the substrate. To account for the scattering we therefore multiply the Fresnel coefficients of the substrate by a Rayleigh scattering factor *s* given by

7 $$\begin{aligned} s = \left( \frac{4\pi D_f }{\lambda } (n_s-1)\right) ^2, \end{aligned}$$

where $\lambda$ is the THz wavelength and $D_f$ is the diameter of the fibre^24,25^. We find for both samples that if the Rayleigh scattering is taken into account then a constant conductivity is able to describe the measured transmission amplitudes well. This extension to Eq. (4) is denoted by ’TMM and Rayleigh scattering’ in the figure. The best fits were obtained for a fibre diameter $D_f$ of 12 μm for both samples. Microscopic images of the substrate revealed a diameter of the individual fibres making up each macroscopic strand of the fabric to be approximately 9 μm. We attribute the difference to the fitted value to the fact that the Rayleigh model is an approximation of the actual scattering behavior of the fibres.

The fitting procedure described above results in a conductivity of ${116\pm 43}\,\hbox {kS}\,\hbox {cm}^{-1}$ and ${1.1\pm 0.3}\,\hbox {kS}\,\hbox {cm}^{-1}$ for the Ag and ITO coated samples respectively. We estimated the uncertainty based on a 50 nm uncertainty in the layer thickness. However the DC conductivity value of pure bulk Ag is ${629}\,\hbox {kS}\,\hbox {cm}^{-1}$ while for a pure ITO film a value of ${10}\,\hbox {kS}\,\hbox {cm}^{-1}$ has been reported^26,27^. It is known that in the case of thin films there are several charge carrier scattering mechanisms that result in a decreased conductivity compared to the bulk conductivity. These effects include scattering at grain boundaries, defects, and interfaces^28,29^. Additionally, due to the negative trend of the real part of the conductivity predicted by the Drude model we generally expect conductivity results based on high frequency measurements to be lower compared to the results obtained through DC measurements. Around ${550}\,\hbox {GHz}$ we found a larger difference between the model and the experimental curves. We attribute this deviation to diffraction effects caused by the larger macroscopic mesh structure of the substrate. A microscopic image of the substrate shown in the top right corner of Fig. 2b) revealed a period of around ${0.5}\,\hbox {mm}$ which approximately corresponds to the wavelength range where we observed the deviation. In^10^ a constant conductivity value of ${460}\,\hbox {kS}\,\hbox {cm}^{-1}$ for a 86 nm thin Ag film is reported. In our opinion, there are various reasons that contribute to the lower conductivities that we measure in comparison to the bulk and even other think film reports in the literature. Firstly, additional defects in the thin film are caused by impurities originating from the substrate. Secondly, the structure of the textile produces local discontinuities in the film, which are absent on similar films deposited on flat substrates. Thirdly, during the coating procedure the textile out-gases into the vacuum which the substances released are subsequently incorporated into the thin film, which in turn, act as defects lowering the conductivity.

**Figure 3:**
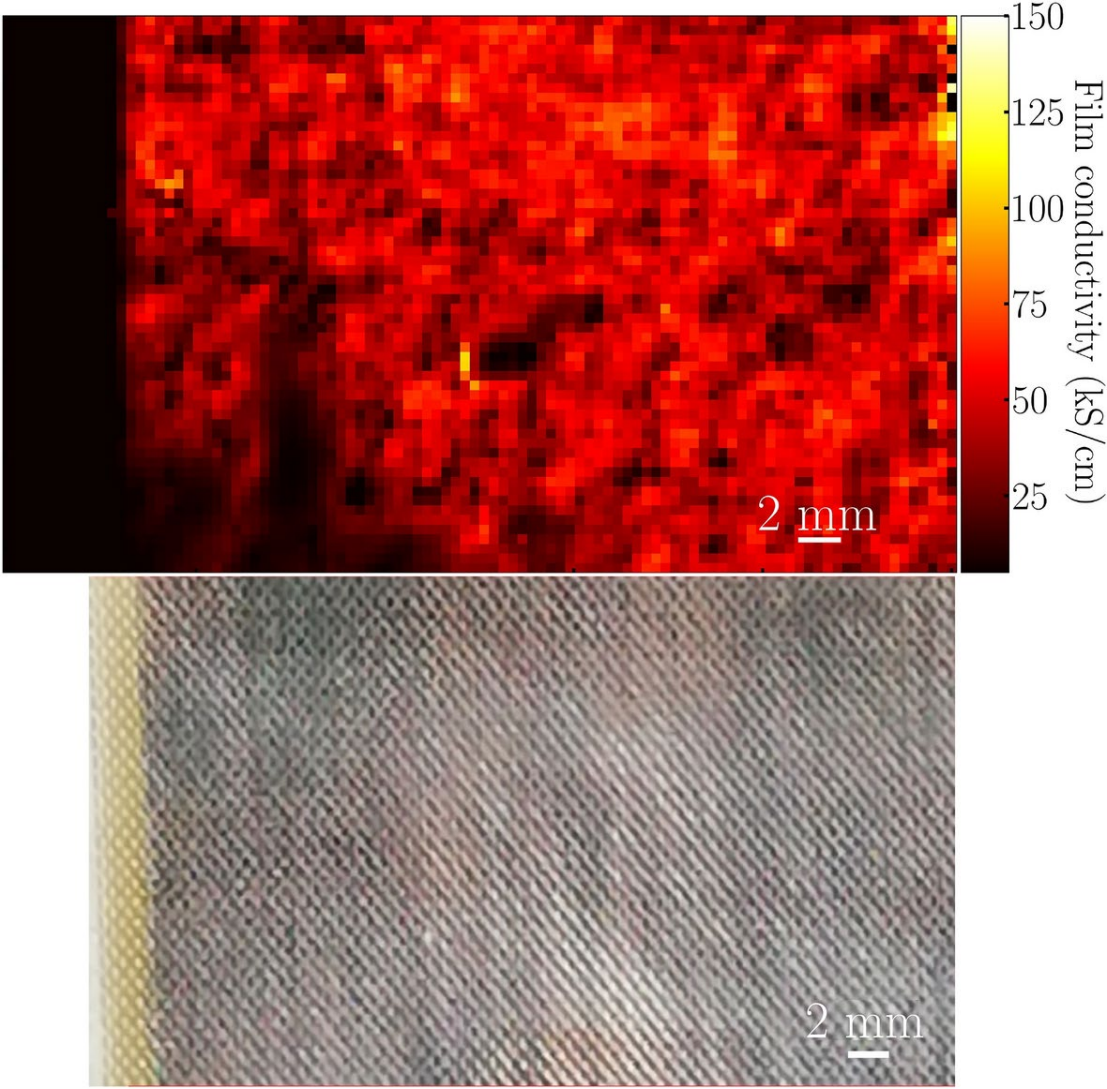
Top part of the figure: Image of the conductivity of the Ag film. Lower part of the figure: Photo of the imaged area. The dark area to the left indicates the edge of the sample.

**Figure 4:**
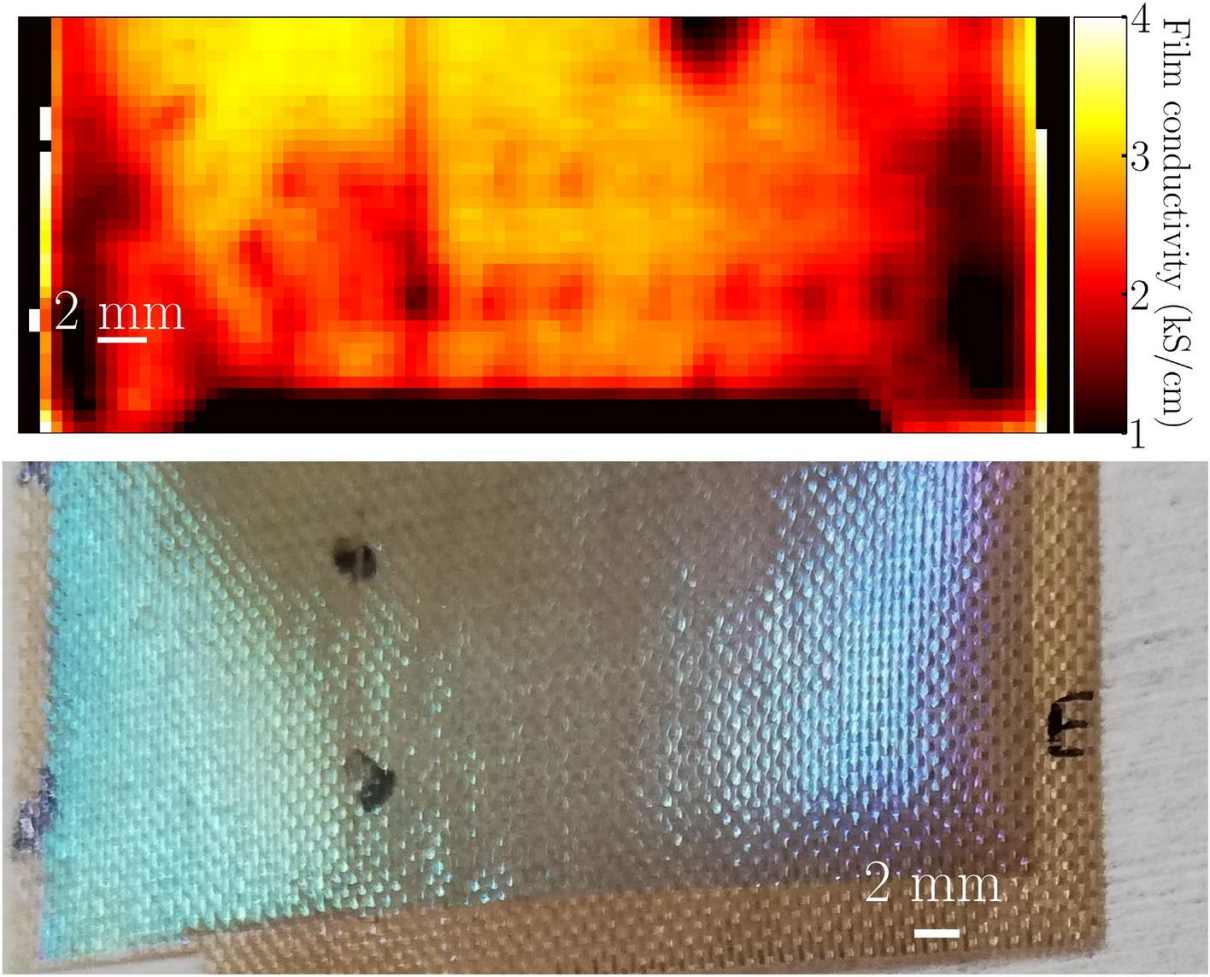
Top part of the figure: Image of the ITO film conductivity. Left to right edge of the image covers the full $5\,\hbox {cm}$ sample width. Lower part of the figure: A photo which highlights the different coloring at the left and right edges of the sample.

To obtain this map reflecting the homogeneity of the film conductivity we fit the transmission given in Eq. (4) to the measured transmission at ${1.2}\,\hbox {THz}$ for the free parameter sigma of the film refractive index and each imaged pixel. The resulting images for the Ag and ITO samples are shown in Figs. 3 and 4, respectively.

In the lower left quarter of the conductivity image shown in Fig. 3 we see two larger areas with lower conductivity, one extending from the edge towards the centre and the second area is located in the centre of the image. These two areas appear similar in the visible image. While for the ITO sample shown in Fig. 4, a lower conductivity at the left and right edges of the imaged area can be observed. These two areas match the iridescent regions of the corresponding visible image, suggesting that the thickness of the film there might be smaller, probably closer to 150 nm instead of 200 nm which is consistent with the variations observed by the Tencor P-7 Stylus Profiler on the flat “witness” samples. Since the fitting does not include the thickness as a free parameter, and given that we do not have an independent method to map out the layer thickness in the coated textile samples, this reflects as a variation of the extracted conductivity.

**Figure 5:**
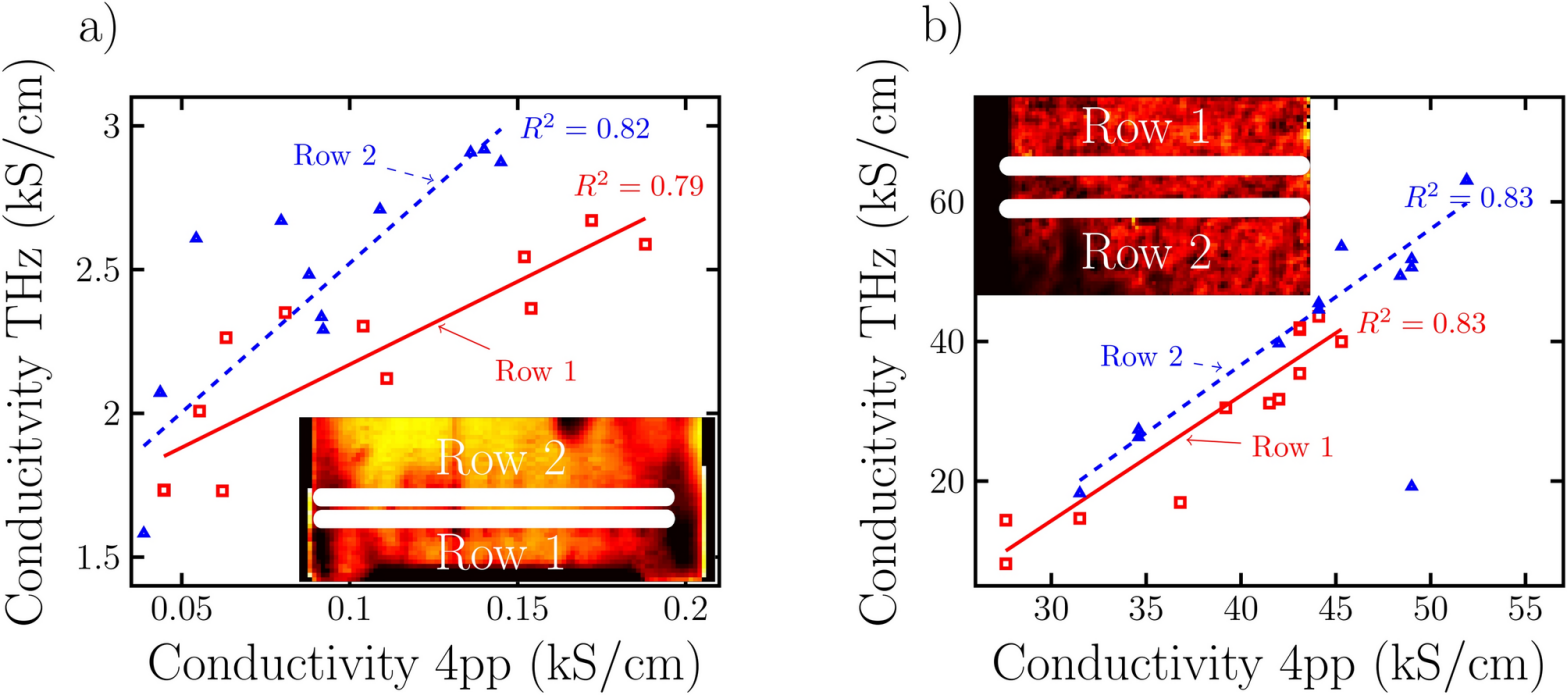
The result of the 4-point probe measurement plotted against the values of the THz conductivity measurement show a strong correlation for both the ITO (**a**) and Ag (**b**) samples.

To confirm the THz conductivity image of the samples we performed four-point probe measurements along some lines on the samples surface for both samples as indicated on the insets to Fig. 5. Each row is divided equally into 12 line segments. In order to correlate the THz and four-point probe measurements we averaged the THz conductivities over these ${3.5}\,\hbox {mm}$ wide line segments. For each four-point probe measurement value we thereby obtained approximately one equivalent THz measurement value. Figure 5 shows the averaged THz conductivity values plotted against the four-point probe values. The lines indicate linear fits of the THz conductivity values as a function of the four-point probe values. Additionally, the coefficient of determination values $\left( R^2\right)$ of the fits indicate a strong correlation between the two measurement techniques for both samples.

Generally for all rows and both samples we find that the four-point probe conductivity is lower compared to the measured THz conductivity. This can be explained by the fact that the conductive layers are not single crystals. Furthermore, since the DC voltage is applied for a longer duration in the case of the four-point probe measurement, the charge carriers will travel a longer distance in the film compared to the THz measurement. This is in contrast to the THz measurement where the general transport length of the current induced by a single cycle of the THz field is on the order of 10 nm to 100 nm^28^. The moving charge carriers will therefore encounter more defects in the case of the four-point probe measurement. Additionally, this indicates the presence of defects or micro tears in the film which are larger than the maximum THz transport length. A similar result was observed in the case of conductivity maps of graphene where it analogously was found that larger defects had a higher impact on the four-point probe measurement^30^.

## Conclusion

We presented the mapped electrical conductivity of textiles coated with thin ITO and Ag films which made it possible to identify areas with a higher or lower conductivity. Furthermore, we found that a constant conductivity model combined with a loss factor which accounts for the scattering of the electromagnetic radiation was able to fit the measured amplitude transmission well. Finally, we were able to confirm the conductivity maps of both samples based on four-point probe measurements which showed a strong correlation to the THz measurements. THz promises to be a fundamental tool for the investigation of conductive textiles and for their quality control.

## Data Availability

The datasets used and/or analysed during the current study are available from the corresponding author on reasonable request.
